# State‐Level Medicaid Expansion and Hospital, Federally Qualified Health Center, and Rural Health Clinic Availability

**DOI:** 10.1111/jrh.70159

**Published:** 2026-05-07

**Authors:** Elliott Paintsil, Sebastian Linde

**Affiliations:** ^1^ Division of General Internal Medicine Department of Medicine Medical College of Wisconsin Milwaukee Wisconsin USA; ^2^ Department of Health Policy & Management School of Public Health Texas A&M University College Station Texas USA

**Keywords:** access to care, FQHCs, hospitals, Medicaid expansion, RHCs

## Abstract

**Background:**

Medicaid expansion in early adopter states has been linked to reduced uncompensated care and improved financial stability of healthcare organizations; however, less is known about its longer‐term effects, outcomes among later adopters, and differences across rural and urban settings.

**Objective:**

To examine the longer‐term association between state Medicaid expansion and the availability of hospitals, federally qualified health centers (FQHCs), and rural health clinics (RHCs) in states expanding Medicaid in 2014, 2015, and 2016, and to assess differences across rural and urban counties.

**Design:**

We applied difference‐in‐differences methods for staggered policy adoption. Covariates included county population, median age, median household income, and racial/ethnic composition. Outcomes were the county‐level total number of hospitals, FQHC sites, and RHC sites, analyzed for all counties and rural/urban subsamples.

**Setting:**

U.S. counties, during 2010–2019 period.

**Participants:**

Two thousand eight hundred and fourteen counties (1319 expansion; 1495 nonexpansion) across 44 states.

**Results:**

Medicaid expansion was associated with slightly more hospitals (0.094; 95% CI –0.003 to 0.190; *p* <0.1) and FQHC sites (0.511; 95% CI 0.214–0.807; *p* <0.01), but no significant change in RHC sites (–0.032; 95% CI –0.310 to 0.247). Relative to 2013, these correspond to county‐level increases of 4.9% for hospitals and 26.7% for FQHCs. Effects appeared in both rural and urban areas, with larger relative gains in rural counties. Event‐study plots indicated that effect sizes increased over time.

**Conclusion:**

Counties exposed to Medicaid expansion experienced sustained growth in hospital and FQHC presence, but not in RHCs, possibly reflecting supply side constraints. Policymakers should recognize that stabilization effects may strengthen gradually following expansion.

## 1 Introduction

Medicaid expansions across several states have been associated with increased access to care, leading to improved health outcomes [1, 2, 3, 4, 5, 6, 7, 8, 9, 10, 11, 12, 13]. Prior work has also shown that Medicaid expansion is associated with families self‐reporting increased financial security and stability [14, 15, 16, 17, 18]. Therefore, Medicaid expansion has shown impact across several social determinants of health domains, providing underserved populations with additional support toward improved quality of life.

The literature further indicates that states with early Medicaid expansion adoption saw significant improvement to the financial stability of healthcare organizations [19, 20, 21, 22, 23, 24]. In particular, prior work documents reductions of hospital closures within rural communities, along with improved financial sustainability upon federally qualified health centers (FQHCs) and upon rural health clinics (RHCs) [5, 25, 26, 27, 28, 29, 30]. The effects of Medicaid expansion on the healthcare system, from both the health system standpoint as well as the patient standpoint, showcase the impact that health insurance coverage boosting policies can have on multiple levels of the healthcare system.

Our study seeks to extend the findings of previous work by examining several Medicaid expansion waves to determine the long‐term impact the policy had on the availability of several health system resources. Specifically, we contribute to the preceding literature by investigating the long‐term association between Medicaid expansion adoption and county level presence of hospitals, FQHC sites, and RHC sites, over the period of 2010 through 2019, which encompasses multiple Medicaid expansion waves affecting both rural and more urban communities. We hypothesize that Medicaid expansion will be associated with increased availability of hospitals, FQHC sites, and RHC sites, with a stronger impact in rural communities, which have traditionally had higher closure rates. We explore our hypothesis utilizing three different methods. First, we study the effects of Medicaid expansion on healthcare organizations across the mainland United States, along with analyses across rural and urban counties. This allows us to examine potential effects of Medicaid expansion policies overall in the United States, as well as across rural and urban settings. Second, we utilize methods that allow us to assess multiple waves of Medicaid expansions (i.e., expansions across 2014, 2015, and 2016). This allows us to examine the effects of Medicaid expansion in states that did not adopt the policy changes in the initial expansion year of 2014. Third, our methods allow us to examine associations between Medicaid expansion adoption and the presence of healthcare organizations over time, something that allows us to examine whether there are temporal lags to the realization of Medicaid expansion effects—insights that are of policy‐making interest and significance.

## 2 Research Design and Methods

### 2.1 Study Samples

State Medicaid expansion status was obtained from the Kaiser Family Foundation Medicaid expansion tracker [31]. Other data were sourced from the AHRQ SDOH database [32], which obtained our main analytic variables from the Provider of Service data from the U.S. Department of Health and Human Services, Centers for Medicare & Medicaid Services; the Area Health Resource Files from the Health Resources and Services Administration; and the American Community Survey data from the U.S. Department of Commerce, U.S. Census Bureau. Data elements were at the county level and cover the 10‐year period of 2010 through 2019. This period was chosen out of consideration for confounding effects due to the COVID19 pandemic. Our final analytic sample consisted of 2814 counties (1319 with exposure to Medicaid expansion; and 1495 with no exposure) across 44 out of 48 mainland U.S. states. We omit Virginia and Maine due to their expanding Medicaid within the last year of our sample; and we also omit California and Minnesota as these states had changes to their Medicaid eligibility prior to 2014 (please see Supplementary Appendix, Figure S1, for details on our final state sample).

### 2.2 Outcome Measures

Our outcome measures consist of three total count measures. The first is the total number of hospitals, where hospitals are defined as a facility that provides medical or surgical care for acute illnesses or conditions. The second is the total number of FQHC service sites, where FQHC service sites are defined as sites by community‐based providers that meet federal eligibility criteria to serve underserved populations and receive funding support under HRSA's Health Center program. The third outcome measure is the total number of RHC service sites, where RHC service sites are defined as outpatient clinic sites located in a designated rural and medically underserved areas that participates in the Medicare/Medicaid Rural Health Clinic program.

### 2.3 Exposure Measure

The exposure measure was a binary indicator variable taking on the value of 1 if a county belonged to a state that had adopted Medicaid expansion in that or prior years, and zero otherwise. That is, the exposure measure remains zero across all years for states that never expand Medicaid during the sample period, and takes on a value of zero in pre‐Medicaid expansion years and a value of 1 in expansion and post‐expansion years for states that adopt Medicaid expansion during the sample period.

### 2.4 County Controls

Controls consisted of the county population; median age, calculated across the total population; the median household income (in dollars), calculated across households; the percentage of population reporting black or African American Race alone in each county, calculated across the total population; the percentage of population reporting Hispanic ethnicity in each county, calculated across the total population; and the percentage of population reporting White race alone in each county, calculated across the total population.

### 2.5 Rurality Measure

To designate the rurality of counties, we drew on rural‐urban continuum codes (RUCC) from the U.S. Department of Agriculture [33]. A county was designated rural if it had an RUCC of 4 or greater; and it was designated as urban if it had an RUCC of less than 4. While these definitions are standard within the literature, we note that as the RUCC classify entire counties, this definition results in instances where smaller metropolitan counties are classified as urban, but also contain RHC sites, as these serve rural parts of that county.

### 2.6 Statistical Analysis

Our analyses used the augmented inverse‐probability weighted difference in difference estimator a la Callaway and Sant'Anna (2021). This method was chosen as it is able to accommodate the staggered Medicaid expansion adoption of different states within our data (i.e., across the years of 2014, 2015, and 2016); because it yields doubly robust estimates for the average treatment effects upon the treated (ATET); and because it allows examination of treatment effects over time via ATET aggregation within post‐treatment periods. For all analyses, county covariates consisted of population count, median age, median household income, and population shares across race and ethnicity. The same set of county covariates were used within the propensity score and outcome regression models; and control units consisted of never adopters (i.e., never adopt Medicaid expansion) and the not yet adopters (i.e., places that later adopt a Medicaid expansion policy, but that have not yet done so). County level outcome measures consisted of the total number of hospitals, total number of FQHC sites, and total number of RHC sites. Analyses were performed across a full sample; and also, across rural (counties with RUCC of 4 or greater) and urban (counties with RUCC of less than 4) subsamples. Analyses were performed using Stata V.19 (StataCorp LLC.), and statistical significance was noted at levels of *p* <0.01, *p* <0.05, and *p* <0.1.

## 3 Results

The analysis sample included 2814 counties (1319 with exposure to Medicaid expansion; and 1495 with no exposure) across 44 mainland U.S. states; and spanned the 10‐year period of 2010 through 2019. The total number of county‐year observations included in the analyses was 28,128. Across U.S. counties, the mean (SD) number of hospitals was 1.92 (3.67) per county, with a range of 0–75. The mean number of FQHC sites was 2.25 (6.19) per county, with a range of 0–236. The mean number of RHC sites was 2.99 (3.54) per county, with a range of 0–32 Table 1.

The Medicaid expansion indicator had a mean (SD) of 0.27 (0.44), corresponding to 27% of our county‐year observations being classified as expansion adoption observations. Across all counties, the median age of the sample was 40.7 years (SD 5.15; range 22.5−67.4). The median household income was $46,768 (SD $12,000; range $18,972−$121,324). Across all counties, the mean (SD) share of residents self‐identifying as non‐Hispanic White was 84.2% (16.2), with values ranging from 3.55% to 100%. The mean share of residents self‐identifying as non‐Hispanic Black was 9% (14.6%), with values ranging from 0% to 87.4%. The mean share of residents self‐identifying as Hispanic was 8.68% (13.53%), with values ranging from 0% to 99.2%. (Additional sample and county descriptives are provided within the Supplementary Appendix, Figure S1, and Tables S1 and S2).

### 3.1 Staggered Difference in Difference ATET Estimates

Counties with exposure to state Medicaid expansion adoption were associated with slightly more hospitals (0.094; 95% CI: –0.003 to 0.190; *p* <0.1) and more FQHC sites (0.511; 95% CI: 0.214–0.807; *p* <0.01), but there were no significant effects noted upon RHC sites (–0.032; 95% CI: –0.310 to 0.247) (Table 2). Relative to the pre‐expansion period of 2013, these estimates indicate that Medicaid expansion adoption was associated with county level average increases of 4.87% for hospitals and 26.70% for FQHC sites (Table 2).

**TABLE 1:** Summary statistics (*N* = 28,128).

	Mean	SD	Min	Max
**Outcome measures**				
Total number of hospitals	1.919	3.673	0	75
Total number of FQHC sites	2.252	6.19	0	236
Total number of RHC sites	2.988	3.541	0	32
**Exposure measure**				
Medicaid expansion indicator	0.267	0.442	0	1
**Control measures**				
Median age	40.739	5.148	22.5	67.4
Median HH income	46,768.454	12,000.366	18,972	121,324
Share NHW	84.182	16.194	3.55	100
Share NHB	9.009	14.613	0	87.41
Share Hispanic	8.678	13.525	0	99.18

Abbreviations: *N*, number of observations; SD, standard deviation.

**TABLE 2:** Overall aggregated average treatment effect on treated (ATET) estimates.

Outcome measure	Sample	ATET (95% CI)	2013 mean outcome measure	Percentage change relative to 2013
**Total number of hospitals**	Full	0.094* (−0.003, 0.190)	1.931	4.868%
	Rural	0.057** (0.012, 0.101)	1.042	5.470%
	Urban	0.158* (−0.019, 0.335)	3.518	4.491%
**Total number of FQHC sites**	Full	0.511*** (0.214, 0.807)	1.914	26.698%
	Rural	0.221* (−0.024, 0.465)	0.926	23.866%
	Urban	0.749*** (0.255, 1.243)	3.678	20.364%
**Total number of RHC sites**	Full	−0.032 (−0.310, 0.247)	2.857	—
	Rural	−0.080 (−0.419, 0.259)	3.295	—
	Urban	0.238* (−0.024, 0.499)	2.076	11.464%

*Note*: Cluster robust 95% CI reported within parentheses. Clustering was done at the state level. Percentage change relative to 2013 is computed using the point estimate ATETs and 2013 mean outcome measures.

Significance indicated as * *p* <0.1, ** *p* <0.05, *** *p* <0.01.

Rural counties with exposure to state Medicaid expansion adoption were associated with more hospitals (0.057; 95% Cl: 0.012–0.101; *p* <0.05) and slightly more FQHC sites (0.221; 95% Cl: –0.024 to 0.465; *p* <0.1); however, similar to the full sample, there were no significant effects noted for RHC sites (–0.080; 95% Cl: –0.419, 0.259). Relative to the pre‐expansion period of 2013, these estimates indicate that Medicaid expansion adoption was associated with rural county level average increases of 5.47% for hospitals and a 23.87% for FQHC sites.

Urban counties with exposure to state Medicaid expansion adoption were similarly associated with slightly more hospitals (0.158; 95% Cl: –0.019, 0.335; *p* <0.1), more FQHC sites (0.749; 95% Cl: 0.255, 1.243; *p* <0.01), as well as with slightly more RHC sites (0.238; 95% Cl: –0.024, 0.499; *p* <0.1). Relative to the pre‐expansion period of 2013, these estimates indicate that Medicaid expansion adoption was associated with urban county level average increases of 4.5% increase in hospitals, 20.36% for FQHC sites, and 11.47% for RHC sites.

**FIGURE 1:**
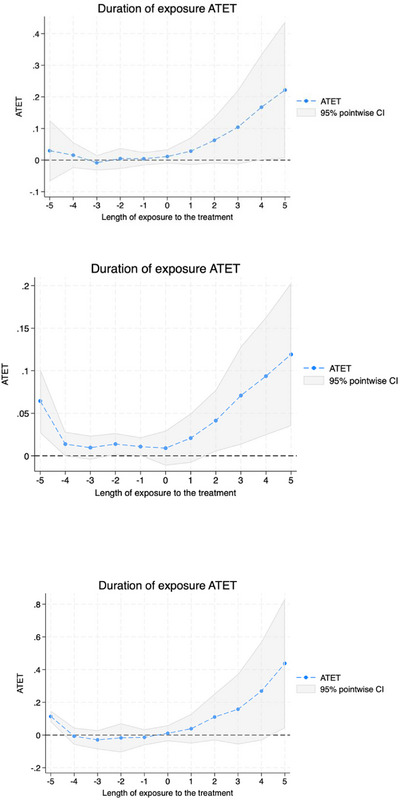
ATET plot for total number of hospitals model. Top is that for full sample; middle for rural subsample; and bottom for urban subsample.

**FIGURE 2:**
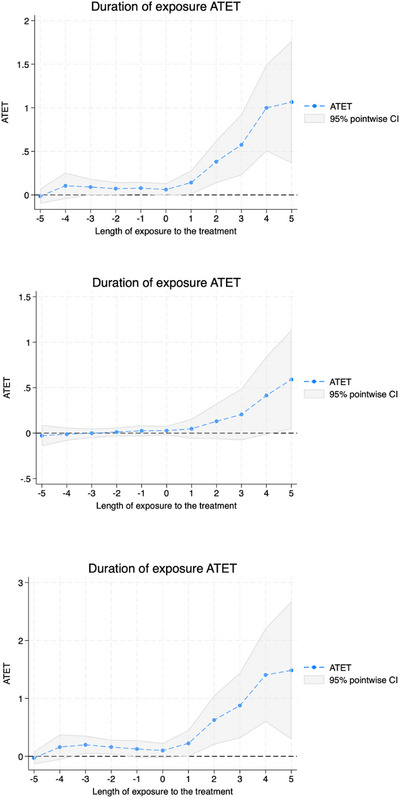
ATET plot for total number of FQHC sites model. Top is that for full sample; middle for rural subsample; and bottom for urban subsample.

**FIGURE 3:**
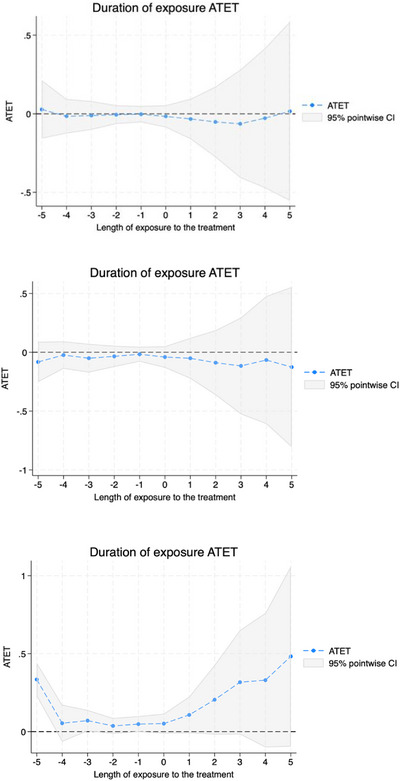
ATET plot for total number of RHC sites model. Top is that for full sample; middle for rural subsample; and bottom for urban subsample.

Figures 1, 2, 3 depict the average treatment effect on treated estimates, where the x‐axis is length of exposure to the treatment in years (where 0 is the initial year of Medicaid expansion adoption; and negative numbers indicate pre‐treatment years) for the total hospitals (Figure 1), total FQHC sites (Figure 2), and RHC sites (Figure 3). The top graph in each figure is the trend for the full sample; the middle is for the rural subsample; and the bottom is for the urban subsample. The positive trend depicted in each graph as we move forward in time from Year 0 of exposure (i.e., Medicaid expansion adoption) further indicates that Medicaid expansion has shown to have a continuing positive effect on total hospitals and FQHC sites with time following a state's Medicaid expansion adoption. The null effect within pre‐treatment years (close to the timing of the policy adoption) also helps provide support for the standard parallel pre‐treatment trends assumption (between adopters and controls), overall, being satisfied by the models.

## 4 Discussion

Our analysis demonstrates that counties with exposure to state Medicaid expansion adoption were associated with relatively more hospitals and FQHC sites, with effects less clear for RHC sites. These findings are consistent with Lindrooth et al., who found fewer rural hospital closures in expansion states through 2016, as well as findings by Behr et al., who found that Affordable Care Act (ACA) funding allowed for FQHC programs to expand access into areas with higher rates of poverty and uninsurance, with less clear effects in rural counties. Our study complements these findings by demonstrating that counties within Medicaid expansion states saw average increases in hospitals and FQHC sites, with associations holding across both rural and urban settings. However, the noted relative gains were larger within rural settings for both hospitals and FQHC sites. Event plot analyses of estimated average treatment effects upon the treated further show that Medicaid expansion effects can take time to materialize, but grow in their effect size with time following states’ Medicaid expansion adoption. These findings emphasize the positive impact of Medicaid expansion adoption on the growth of a state's healthcare system at the county level, regardless of urbanity or rurality. It also highlights that such effects may take time to fully materialize.

Current policy proposals, however, indicate that many may come to lose Medicaid coverage. The literature shows this will have a lasting effect on insurance coverage and the health of patients [1, 2, 3, 4, 5, 6, 7, 8, 9, 10, 11, 12, 13]. In particular, prior work done by Luo et al., and Jiao et al., demonstrated the adverse financial impacts of a state's decision to not participate in or repealing Medicaid expansion by showing improved financial stability and ability to accommodate a higher patient census across states that received increased funding post‐ACA adoption. Our study complements this literature by showing that Medicaid expansion is associated with increases in FQHC service sites, and not solely the volume of patients served. As such, policymakers ought to consider the broad impacts of Medicaid expansion policies across effects on covered individuals as well as upon the well‐being of fragile U.S. healthcare markets that have seen broad closures across both rural and urban counties in the United States.

### 4.1 Study Limitations

This study utilized an observational study design, and as such, although the methods are quasi‐experimental, the results are indicative of associations and not causal effects. Our data came from across 44 mainland U.S. states, and as such, findings may not generalize beyond these states. We utilize RUCC to classify entire counties as either urban or rural. While this classification approach is standard within the literature, this classification is imperfect as this definition may result in instances where smaller metropolitan counties are classified as urban, but also contain RHCs as these serve rural parts of that county.

## 5 Conclusion

We find that counties with Medicaid expansion exposure experience increased stabilization and growth within their hospital and FQHCs presence, but do not experience similarly consistent trends pertaining to RHCs. This may reflect supply side constraints that may be disproportionately experienced by RHCs. Additionally, policymakers looking to use Medicaid expansion as a lever for market stabilization should be mindful that such stabilization may take time to fully materialize.

## Conflicts of Interest

The authors declare that they have no conflicts of interest.

## Funding

No financial disclosures are reported by the authors of this paper.

## Supporting information




**Supporting File 1**: jrh70159‐sup‐0001‐SuppMat.docx
