# Supplementary material for: State‐Level Medicaid Expansion and Hospital, Federally Qualified Health Center, and Rural Health Clinic Availability
Source: J Rural Health. 2026 May 7;42:e70159. doi: 10.1111/jrh.70159 (PMC13150399; doi:10.1111/jrh.70159)
Supplement: Supplementary file 1 — Supporting File 1: jrh70159‐sup‐0001‐SuppMat.docx [file JRH-42-0-s001.docx]

**Supplemental Online Appendix**

**Appendix A – States Included within Analytic Sample; Unique Counties by State; and Summary Statistics by Expansion Status in 2013.**

Figure S1 indicates our final analytic state sample, which excluded states with (1) changes to their Medicaid eligibility criteria prior to 2014 (California and Minnesota), and (2) those with expansion within our final sample year of 2019 (Virginia and Maine). Table S2 further documents the number of unique counties by state, while Table S3 provides descriptives by expansion status (Medicaid expansion non-adopters vs. Medicaid adopters).

**Figure S1: States Included within Analytic Sample**

**Table S1: Number of Unique Counties by State**

| State | Number of Unique Counties |
| --- | --- |
| Alabama | 67 |
| Arizona | 15 |
| Arkansas | 75 |
| Colorado | 64 |
| Connecticut | 8 |
| Delaware | 3 |
| Florida | 67 |
| Georgia | 159 |
| Idaho | 44 |
| Illinois | 102 |
| Indiana | 92 |
| Iowa | 99 |
| Kansas | 105 |
| Kentucky | 120 |
| Louisiana | 64 |
| Maryland | 24 |
| Massachusetts | 14 |
| Michigan | 83 |
| Mississippi | 82 |
| Missouri | 115 |
| Montana | 56 |
| Nebraska | 93 |
| Nevada | 17 |
| New Hampshire | 10 |
| New Jersey | 21 |
| New Mexico | 33 |
| New York | 62 |
| North Carolina | 100 |
| North Dakota | 53 |
| Ohio | 88 |
| Oklahoma | 77 |
| Oregon | 36 |
| Pennsylvania | 67 |
| Rhode Island | 5 |
| South Carolina | 46 |
| South Dakota | 66 |
| Tennessee | 95 |
| Texas | 254 |
| Utah | 29 |
| Vermont | 14 |
| Washington | 39 |
| West Virginia | 55 |
| Wisconsin | 72 |
| Wyoming | 23 |

**Table S2: Summary Statistics in 2013 by Medicaid non-Expansion and Expansion Counties.**

|  | Mean | SD |
| --- | --- | --- |
| **Medicaid non-Expansion Counties in 2013 (n=1,494)** |  |  |
| Share Rural | 0.664 | 0.473 |
| Median Age | 39.991 | 5.256 |
| Median HH Income | 43,173.406 | 9,653.998 |
| Population Count | 364,825.030 | 1,042,956.500 |
| Share NHW | 81.184 | 17.793 |
| Share NHB | 11.972 | 17.02 |
| Share Hispanic | 10.281 | 15.315 |
| **Medicaid Expansion Counties in 2013 (n=1,319)** |  |  |
| Share Rural | 0.615 | 0.487 |
| Median Age | 41.162 | 4.754 |
| Median HH Income | 47121.932 | 12193.719 |
| Population Count | 558,310.77 | 1,436,915.000 |
| Share NHW | 88.184 | 13.032 |
| Share NHB | 5.563 | 10.226 |
| Share Hispanic | 6.457 | 10.555 |
